# Nutrition Standards for Food Service Guidelines for Foods Served or Sold in Municipal Government Buildings or Worksites, United States, 2014

**DOI:** 10.5888/pcd13.160364

**Published:** 2016-12-22

**Authors:** Stephen J. Onufrak, Hatidza Zaganjor, Latetia V Moore, Susan Carlson, Joel Kimmons, Deborah Galuska

**Affiliations:** 1 Centers for Disease Control and Prevention, Division of Nutrition, Physical Activity and Obesity; Atlanta, GA 30341.

## Abstract

**Introduction:**

The Institute of Medicine and Centers for Disease Control and Prevention have recommended that government agencies use nutrition standards for foods and beverages sold and provided at their facilities. In this study, we examine written nutrition standards for foods sold or served in local government buildings or worksites among US municipalities.

**Methods:**

We used data from a 2014 national survey of 1,945 municipal governments serving populations of 1,000 or more to assess the presence of written nutrition standards, the food groups or nutrients addressed by standards, and the populations served by facilities where standards are applied. The prevalence of standards was estimated by municipality population size, rural–urban status, census region, poverty prevalence, education level, and racial/ethnic composition.

**Results:**

Overall, 3.2% of US municipalities reported nutrition standards with greater prevalence observed among large municipalities (12.8% of municipalities with ≥50,000 people vs 2.2% of municipalities with <2,500 people, *P* < .001). Prevalence differed by region, and standards were most common in the West (6.6%) and least common in the Midwest (2.0%, *P* = .003).The most common nutrition topics addressed in standards were offering low-calorie beverages, fruits and vegetables, and free drinking water. Most standards applied to facilities serving government employees (67%) or the general public (66%), with fewer serving institutionalized populations (23%).

**Conclusion:**

Few municipal governments reported having written nutrition standards for foods and beverages sold in their facilities in 2014. Implementing nutrition standards for foods sold or served by local governments is a strategy for increasing access to healthier foods and beverages among municipal employees and local residents.

## Introduction

Food service guidelines (FSGs) delineate food and nutrition standards for the sale or provision of foods and beverages in food venues such as cafeterias and vending machines and may specify approaches such as pricing healthier foods lower than less healthy foods (1). FSG nutrition standards are effective at increasing the availability and purchasing of healthier foods while decreasing the purchasing of less healthful foods (2). The Institute of Medicine and Centers for Disease Control and Prevention (CDC) have recommended that government agencies use nutrition standards for foods and beverages that they sell or provide (3–5). More than 10 million people in the United States are employed by local governments (6), so enactment of FSGs among municipalities can improve food environments in government facilities serving employees, local residents, and institutionalized people.

Although information on some nutrition standards for local governments are available (7), their prevalence among US municipalities, populations affected, and nutritional characteristics of the standards are not known. This information would be useful for tracking local government efforts to improve the availability of healthier foods over time. However, capturing this information is difficult, because there are approximately 19,500 incorporated places in the United States (8), and municipal policies are not systematically captured by legal databases. To fill information gaps on local health policies, the CDC Division of Nutrition, Physical Activity, and Obesity conducted the National Survey of Community-Based Policy and Environmental Supports for Healthy Eating and Active Living (CBS HEAL) to query municipal officials about the presence of local health policies and practices. In this study, we use data from CBS HEAL to determine the national prevalence among US municipalities of written nutrition standards, prevalence differences according to municipality characteristics, and the nutrition characteristics and populations covered by the standards.

## Methods

### Survey and analytic sample

The CBS HEAL survey of 4,484 municipalities from all 50 states was conducted from May 2014 through September 2014 and had a response rate of 45% (n = 2,029). The sample pool of potential respondents was based on the 2007 Census of Governments, which lists municipalities and townships by state and provided the most recent data available at the time of sampling (9). In states where there was geographic overlap between municipal and town or township levels of government, towns and townships were removed to avoid double counting of populations covered by both layers of government. To create a nationally representative sample of municipalities, sampling was stratified by region, urban status, and population size, and the observations were weighted to account for unequal probabilities of selection and varying rates of nonresponse. Municipalities with populations smaller than 1,000 were excluded from the sample pool on the basis of a previous pilot study that showed that very small communities were not likely to have policies and practices that support healthy eating and active living. The primary survey respondent was the city or town manager, planner, or person with similar responsibilities. Respondents were encouraged to ask for assistance if needed from other municipal officials such as representatives from tax office or procurement departments, parks and recreation departments, or human resources. Respondents completed the survey through a secure website and were also given the option of completing a paper version of the survey.

### Variables

Respondents were asked “Does your local government have written nutrition standards for foods sold or served in local government buildings or worksites, including meals, á la carte items, or vending machines? Examples of nutrition standards include provisions for reduced sodium content or for inclusion of fresh fruit and vegetable selections. Do not include public school district or school-level policies in your response.” Respondents who answered yes were asked if the standards included the following nutritional elements, which are consistent with the Health and Sustainability Guidelines for Federal Concessions and Vending Operations (10): vegetables and fruits, whole-grain options, low-fat dairy, low-sodium items, only offering foods with no *trans* fat, labeling foods with calories per serving, lower-calorie beverage choices, or free drinking water. Respondents who reported having standards were also asked if standards applied to government facilities that serve foods and beverages to each of the following groups: local government employees, the general public, or institutionalized people (for example, prisons, centers for the disabled). Because pricing incentives to encourage healthier food sales are encouraged in the Health and Sustainability Guidelines for Federal Concessions and Vending Operations (10), all respondents were asked “Does your local government have pricing incentives to promote the purchase of healthier foods and beverages sold in local government buildings, including cafeterias or vending machines? An example of this is intentionally pricing more healthful items to be less expensive. Do not include public school district or school-level policies in your response.”

Other variables used in this analysis included municipal population size, rural–urban status, region, median educational attainment, poverty prevalence, and percentage of residents who were non-Hispanic white. The population size of each municipality was determined with information from the US Census Bureau 2007 Census of Governments (9). For the present analysis, we classified population size into 3 categories: 1,000 to 2,499, 2,500 to 49,999, and 50,000 or more. Municipalities were classified as urban if more than 50% of residents resided in urban areas according to the 2010 US Census Urban Area to Place Relationship File (11). Region was classified according to 4 primary census regions: Northeast, Midwest, South, and West (12). Poverty prevalence was derived from 2009–2013 American Community Survey 5-Year Estimates (13) and dichotomized as either less than 20% or 20% or more on the basis of the established definition of persistent poverty of the US Department of Agriculture (14). Median educational attainment (classified as ≥some college or ≤high school diploma) and racial/ethnic composition of each municipality were obtained from American Community Survey 2009–2013 5-year estimates (13). The racial/ethnic composition of each municipality was classified as more than 90% non-Hispanic white, more than 50% but less than or equal to 90% non-Hispanic white, or 50% or less non-Hispanic white to correspond with municipalities with low percentages of racial/ethnic minority residents, municipalities with mixed populations, or municipalities where minority racial/ethnic groups constituted the majority of residents.

### Statistical analysis

For these analyses, 84 municipalities were excluded because of a missing response (n = 4) or a response of “don’t know” (n = 80) to the nutrition standards survey question. Prevalence among US municipalities of having nutrition standards and healthier food pricing incentives and associated 95% confidence intervals (CIs) were calculated overall and, for nutrition standards, by population size, rural–urban status, region, poverty prevalence, median educational attainment, and racial/ethnic composition. Because few municipalities reported healthier food pricing incentives, it was not possible to examine differences according to municipal characteristics for this variable. Differences in prevalence of nutrition standards according to municipal characteristics were assessed using χ^2^ tests with significance level set at *P* < .05. We also assessed independent associations between the presence of nutrition standards and municipal characteristics by using multivariable logistic regression. The model included nutrition standards as the dependent variable and population size, rural–urban status, census region, educational attainment, poverty prevalence, and race/ethnicity as independent variables. Among municipalities with standards, we also calculated the frequency of each nutrient or food category addressed in the standards and the frequency that the standards applied to government employees, the general public, and institutionalized people. All data analyses were weighted and conducted using SAS version 9.3 (SAS Institute, Inc).

## Results

Among municipalities in the United States in 2014, 3.2% had nutrition standards for foods served or sold in municipal government buildings or worksites (Table). Less than 1% (0.5%; 95% CI, 0.2%–0.9%) had pricing incentives to promote the purchase of healthier foods and beverages.

**Table:** Prevalence of Written Nutrition Standards for Foods Sold or Served in Local Government Buildings or Worksites Among Municipalities, by Municipality Characteristic, United States, 2014

Municipality Characteristic (n)	Yes, % (95% CI)	Odds Ratio^b^ (95% CI)	*P* Value^a^
**All municipalities (n = 1,945)^c^**	3.2 (2.4–4.0)	—	—
**Population size**			
1,000–2,499 (n = 695)	2.2 (1.1–3.3)	1.1 (0.4–2.8)	<.001
2,500–49,999 (n = 1,116)	2.7 (1.8–3.7)	1 [Reference]	
≥50,000 (n = 134)	12.8 (7.0–18.5)	3.0 (1.5–6.2)	
**Rural–urban status**			
Urban (n = 1,423)	3.6 (2.6–4.6)	0.9 (0.3–2.5)	.11
Rural (n = 522)	2.1 (0.9–3.4)	1 [Reference]	
**Census region**			
Northeast (n = 227)	4.0 (1.4–6.6)	1.8 (0.7–4.3)	.003
Midwest (n = 718)	2.0 (0.9–3.0)	1 [Reference]	
South (n = 678)	2.8 (1.6–4.0)	0.8 (0.4–1.7)	
West (n = 322)	6.6 (3.9–9.3)	1.6 (0.7–3.5)	
**Median educational attainment**			
≥Some college (n = 1,083)	3.7 (2.5–4.8)	1.6 (0.8–3.2)	.21
≤High school graduate (n = 862)	2.7 (1.6–3.7)	1 [Reference]	
**Poverty prevalence**			
<20% (n = 1,352)	3.0 (2.1–3.9)	1 [Reference]	.34
≥20% (n = 593)	3.8 (2.3–5.3)	1.2 (0.6–2.2)	
**Race/ethnicity, % non-Hispanic white**			
>90 (n = 720)	1.5 (0.6–2.4)	1 [Reference]	<.001
51–89 (n = 970)	2.9 (1.8–3.9)	1.6 (0.8–3.2)	
≤50 (n = 255)	9.2 (5.7–12.8)	5.6 (2.5–12.6)	

Abbreviations: —, not applicable; CI, confidence interval.

a Calculated by using χ^2^ test.

b Adjusted for population size, rural–urban status, census region, educational attainment, poverty prevalence, and race/ethnicity.

c Total number of survey respondents was 2,029; for this study, 84 municipalities were excluded because of missing or “don’t know” responses to the survey question regarding whether their municipality has written nutrition standards.

Prevalence of nutrition standards differed significantly by population size and were more common among municipalities with populations of 50,000 or more (12.8%) compared with municipalities with fewer than 2,500 (2.2%) or 2,500 to 49,999 (2.7%). After multivariable adjustment, municipalities with populations of 50,000 or more had approximately 3 times greater odds of having nutrition standards compared with those with 2,500 to 50,000 (*P* < .001). Prevalence of standards differed by census region (*P* = .003) with standards most common among municipalities in the West (6.6%) and least common among municipalities in the Midwest (2.0%), but this difference did not remain significant in the multivariable model. Standards were more common among municipalities where non-Hispanic whites composed 50% or less of the population (9.2%) versus municipalities where more than 90% of the population was non-Hispanic white (1.5%) or 51% to 89% was non-Hispanic white (2.9%), and this difference remained significant after adjustment (odds ratio = 5.6 for ≤50% non-Hispanic white compared with >90% non-Hispanic white). Prevalence of standards did not differ according to rural–urban status, educational attainment, or poverty prevalence.

**Figure 1:**
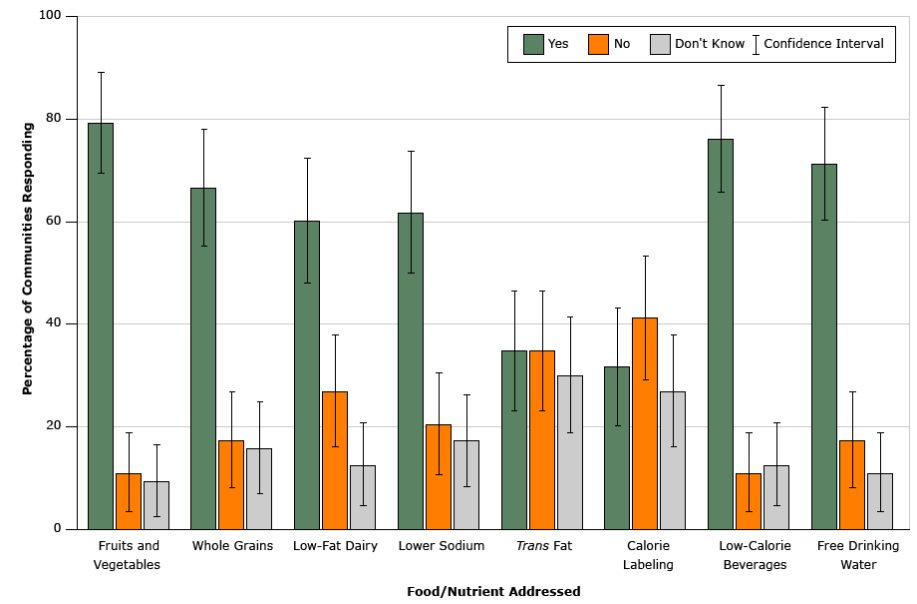
Foods and nutrients addressed in municipal nutrition standards among United States municipalities that reported having them in 2014 (n = 63). National Survey of Community-Based Policy and Environmental Supports for Healthy Eating and Active Living. **Table:** 

Among municipalities that reported having nutrition standards (n = 63), 79% (95% CI, 69%–89%) reported they addressed providing fruits and vegetables, 76% (95% CI: 66%–87%) reported that they addressed providing low calorie beverages, and 71% (95% CI: 61%–83%) reported that they addressed providing free drinking water (Figure 1). Providing whole grains (67%; 95% CI, 55%–78%), low sodium foods (62%; 95% CI, 49%–74%), and low-fat dairy products (60%; 95% CI, 47%–72%) were also reported to be addressed among the majority of municipalities that reported having nutrition standards. Calorie labeling (32%; 95% CI, 21%–44%) and limiting trans-fats (35%; 95% CI: 22%–45%) were reported less frequently.

Food Type or Nutrient	Yes	No	Confidence Interval	Don't Know
	% of Municipalities Responding			
Fruits and vegetables	79.4	11.1	69–89	9.5
Whole grains	66.7	17.5	55–78	15.9
Low-fat dairy	60.3	27	47–72	12.7
Lower sodium	61.9	20.6	49–74	17.4
*Trans* fat	34.9	34.9	22–45	30.1
Calorie labeling	31.8	41.3	21–44	27
Low-calorie beverages	76.2	11.1	66–87	12.7
Free drinking water	71.4	17.5	61–83	11.1

**Figure 2:**
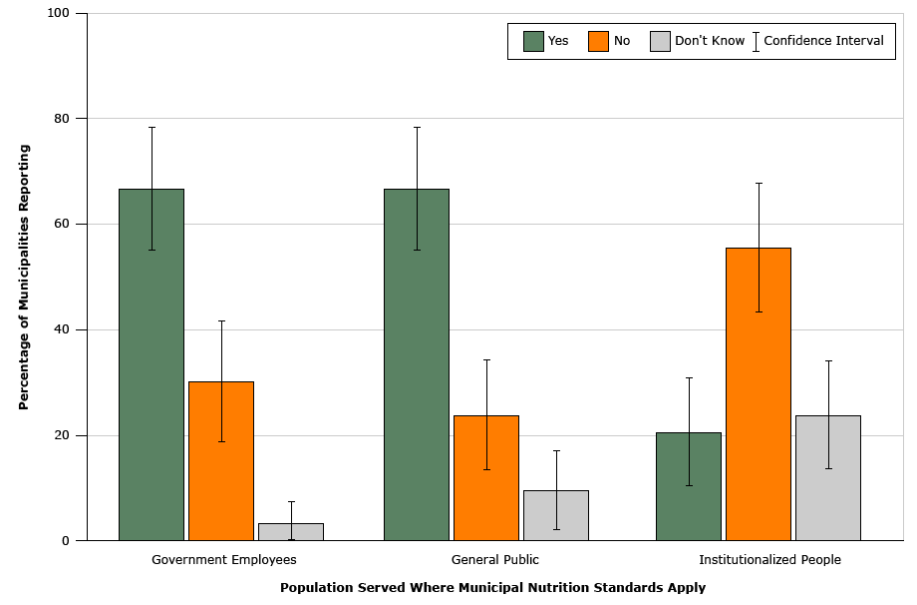
**Reported populations served by facilities in US municipalities that reported having written nutrition standards in 2014 (n = 63).** **National Survey of Community-Based Policy and Environmental Supports for Healthy Eating and Active Living.** **Table:** 

Among municipalities with nutrition standards, 67% (95% CI, 55%–78%) of municipalities reported that standards applied to facilities serving government employees, 67% (95% CI, 54%–78%) reported that they applied to facilities serving the general public, and 49% (95% CI: 36%–61%) reported that standards applied to both employees and the general public. Only 21% (95% CI: 11%–32%) of municipalities with standards reported that they applied to facilities serving institutionalized persons (Figure 2).

Population Served	Yes	No	Confidence Interval	Don't Know
	% of Municipalities Reporting			
Government employees	66.7	30.2	55–78	3.2
General public	66.7	23.8	54–78	9.5
Institutionalized people	20.6	55.6	11–32	23.8

## Discussion

In 2014, only 3% of US municipalities reported having FSG standards and less than 1% reported having pricing incentives for healthier foods. The prevalence of written nutrition standards was significantly greater among large municipalities and among municipalities where most residents were races/ethnicities other than non-Hispanic white. To our knowledge, this is the first nationally representative assessment of nutrition standards and healthy-food pricing incentives among municipalities in the US and provides a baseline for tracking municipal progress toward ensuring healthier foods are available to employees and residents.

Most Americans do not consume a diet that aligns with dietary guidance, and efforts are needed to make doing so easier for Americans (15). Enactment of FSGs at the local level is one strategy for doing this. In the health impact pyramid model described by CDC Director Thomas Frieden, changing the context of people’s surroundings to make default decisions healthier ranks second in potential public health impact only to improving socioeconomic factors such as poverty and education (16). Examples of municipal policies to make default decisions healthier that have improved human health include fluoridation of drinking water, smoke-free workplaces, and community design to improve physical activity (16). Similarly, another strategy for nutrition standards for FSGs is to make default decisions healthier for government employees and members of the public who are served by or purchase food from governments. Until recently, government nutrition standards have largely focused on child care and school standards regarding the healthfulness of school meals and competitive foods (17) and on US military nutrition standards to maintain a healthy weight among personnel, but policies to enact nutrition standards in other government settings are more recent.

Evaluation of FSG nutrition standards for government facilities is ongoing; however, early evidence has suggested nutrition standard policies have resulted in changes to the food environment such as increased availability of healthier foods and beverages (18,19). Although evaluation of sales and dietary intake changes in response to nutrition standards enactment in government facilities in the United States is limited, changes to cafeteria and vending are associated with increasing purchases and consumption of healthier foods in schools, hospitals, and other settings (20–23). Although the use of pricing incentives is rare among municipalities at this time, they have been used successfully to increase the sales of healthier food and drink selections in worksites, parks, and other venues (24).

The prevalence of nutrition standards among US municipal governments may be low, because the idea is new and because recommendations for municipalities to enact nutrition standards are recent. For example, in 2009 CDC recommended that “communities should increase availability of healthier food and beverage choices in public service venues” as a strategy for obesity prevention in the report, Common Community Measures for Obesity Prevention (5). In 2009 the Institute of Medicine recommended that governments enact nutrition standards, especially for facilities serving the public, in the report, Local Government Actions to Prevent Childhood Obesity (4). Establishment of broad FSG nutrition standards in the federal government began in 2011 when the Health and Sustainability Guidelines for Federal Concessions and Vending Operations were released and continues, as they are integrated into concessions and vending operations across the federal government (10,25). Nutrition standards are also recent at the state government level, with the earliest example being vending standards enacted in California in 2007 (7). Some of the earliest examples of local nutrition standards are Los Angeles County’s 2006 Vending Machine Nutrition Policy and New York City’s 2008 Standards for Meals/Snacks Purchased (26). Los Angeles County’s policy set standards for foods and beverages sold in vending machines in all county facilities and offices (26). New York City’s policy set standards for foods purchased and served by city agencies, foods and beverages sold in vending machines, foods served at meetings and events, and foods sold in commissaries in city correctional facilities (26). As more examples of FSG nutrition standards at different levels of government are publicized and disseminated to local public health and government officials along with evaluations of their impact, the prevalence among municipal governments may increase.

Our study showed no differences by rural–urban status, educational attainment, or poverty prevalence, but large municipalities had a greater prevalence of nutrition standards. There are several reasons that could explain this finding. First, the standards may be facilitated by a greater demand for healthier foods among larger populations as well as better access to food distributors who provide healthier items. Second, enacting nutrition standards may take priority in larger cities with larger government structures, more venues to sell foods, and more programs to provide foods to the public or to institutionalized people. For example, nutrition standards in New York City cover approximately 260 million meals and snacks served by the city at more than 3,000 sites each year (27). Third, government structures of larger municipalities have more specialized departments that address a greater breadth of issues such as public health and may be more likely to take part in novel health promotion efforts. As an example, the Big Cities Chronic Disease Community of Practice represents health officials from the 50 largest cities and metropolitan regions in the United States and listed improving healthy nutrition standards and guidelines as a priority in 2015 (28).

We also found that the presence of nutrition standards was more likely among municipalities where non-Hispanic whites represented 50% or less of the residents. Although it is difficult to speculate the reasons for this, cities with large minority populations may have greater motivation to address obesity and chronic diseases through nutritional policies, because these conditions represent a greater burden among many minorities (29). Previous research also suggests that Hispanics and non-Hispanic blacks may be more likely than non-Hispanic whites to support policy measures to increase the availability of healthier foods such as programs to help small food stores stock fresh fruits and vegetables and creation of community gardens (30). Thus, municipal governments with large minority populations may be motivated to enact FSG nutrition standards because of a high burden of chronic diseases as along with increased resident support for nutritional policy measures to address these conditions.

Although our study is the first examination of local government nutrition standards at a national level, it has limitations. First, although we aimed to create a nationally representative sample by using a stratified sample design and applied sample weights to correct for selection probability and nonresponse, our low response rate may have affected the representativeness. Specifically, although nonrespondents did not differ significantly from respondents by population size or urban–rural status, response rates were lower among municipalities in the Northeast and higher among municipalities in the West. Second, we did not survey county governments, which may provide a large share of government services in places where there are few or no government subdivisions below the county level. However, approximately 62% of the US population lived in incorporated places (municipalities) in 2013 (7). Third, we did not ask respondents if foods or beverages were served or offered for sale in government facilities. Therefore, some of the municipalities that reported not having nutrition standards may not serve or sell any foods or beverages and would have little reason to enact standards. Fourth, we did not ask about the specific government departments or facilities where the standards applied or whether they covered cafeterias, vending machines, or other venues. Thus, we are unable to estimate the reach of reported nutrition standards or to better describe the settings where standards are applied. Fifth, we relied on respondent report and did not consult written policy sources to verify the existence of any policies, and we could not examine implementation level.

In summary, our national survey found that nutrition standards were rare for foods sold or served in municipal government facilities in 2014 but were present for approximately 1 in 8 municipalities with populations of 50,000 or more. Given the 10 million people employed by municipal governments in the United States and the large number of public citizens served by these governments (6), the potential impact of nutrition standards among municipal governments is large. Future work should continue to monitor the enactment of nutrition standards among municipal governments and evaluate their effect on sales and consumption of healthier foods and beverages.
